# Expression of cartilage-derived morphogenetic protein in human intervertebral discs and its effect on matrix synthesis in degenerate human nucleus pulposus cells

**DOI:** 10.1186/ar2808

**Published:** 2009-09-15

**Authors:** Christine L Le Maitre, Anthony J Freemont, Judith A Hoyland

**Affiliations:** 1 Biomedical Research Centre, Biosciences, Faculty of Health and Wellbeing, Sheffield Hallam University, City Campus, Owen Building, Howard Street, Sheffield, S1 1WB, UK; 2 Tissue Injury and Repair Group, School of Clinical and Laboratory Sciences, Faculty of Medical and Human Sciences, Stopford Building, The University of Manchester, Oxford Road, Manchester, M13 9PT, UK

## Abstract

**Introduction:**

Loss of intervertebral disc (IVD) matrix and ultimately disc height as a result of 'degeneration' has been implicated as a major cause of low back pain (LBP). The use of anabolic growth factors as therapies to regenerate IVD matrix, hence restoring disc height and thus reversing degenerative disc disease, has been suggested. Cartilage-derived morphogenetic protein (CDMP) is a growth factor which stimulates proteoglycan production in chondrocyte-like cells and thus could be a useful growth factor for LBP therapies. However, little is known about the expression of CDMP or its receptor in human IVD, nor its effects on human disc cells.

**Methods:**

Using immunohistochemistry we investigated the localisation of CDMP in non-degenerate and degenerate human IVDs. Additionally, we investigated the effect of CDMP on aggrecan and type II collagen gene expression and proteoglycan synthesis in nucleus pulposus (NP) cells derived from degenerate IVDs.

**Results:**

We demonstrated that CDMP 1 and 2 were expressed in the non-degenerate and degenerate IVD, particularly in cells of the NP. A small decrease in the number of CDMP 1 and 2 immunopositive cells was seen with degeneration. Treatment of human NP cells, (derived from degenerate IVD), with CDMP showed an increase in aggrecan and type II collagen gene expression and increased production of proteoglycan (GAGs).

**Conclusions:**

The data suggests that CDMP may be a useful growth factor to stimulate proteoglycan production in the human degenerate IVD and hence the repair of the extracellular matrix.

## Introduction

Low back pain (LBP) is a major problem in the western world, affecting approximately 11 million people in the UK for at least one week each month [1]. It leads to a considerable loss of working days and has a significant impact on the national health service [2]. Imaging studies indicate a link between degeneration of the intervertebral disc (IVD) and LBP [3,4]. However, current conservative and invasive interventions for IVD degeneration, aimed at improving LBP, are only directed towards symptomatic relief. Currently, there are few treatments aimed at repairing the degenerate IVD, which if developed could not only relieve symptoms but prevent their reoccurrence through restoration of normal IVD structure and function. Modern advances in therapeutics, particularly cell and tissue engineering, offer potential methods for inhibiting or reversing IVD degeneration that have not previously been possible. However, to ensure success they require a greater level of understanding of the pathobiology of IVD degeneration than is currently available [5].

The IVD is composed of a proteoglycan rich nucleus pulposus (NP), which is constrained by the surrounding annulus fibrosus (AF) and cartilaginous endplates. During IVD degeneration there is a change in cell phenotype resulting in decreased matrix production, particularly proteoglycan synthesis, and an increase in degradation of IVD matrix by locally produced matrix metalloproteinases (MMPs) and ADAMTS (a disintegrin and metalloprotease with thrombospondin motifs) [6,7]. The overall loss of normal disc matrix results in decreased weight bearing capacity, leading to the generation of fissures, annular tears and the generation of pain.

Several studies have suggested the use of anabolic growth factors to regenerate the matrix of the IVD and hence restore disc height, thereby reversing degenerative disc disease. Numerous growth factors have been implicated and those that have attracted the most attention include transforming growth factor (TGF), insulin-like growth factor (IGF), bone morphogenetic proteins (BMPs), cartilage derived morphogenetic proteins (CDMPs) and fibroblast growth factor (FGF). All these factors have been investigated in *in vitro *studies together with some *in vivo *animal studies, and due to their ability to stimulate the synthesis of matrix components of the IVD, (particularly proteoglycans), have been postulated to be therapeutic agents for the restoration of IVD matrix [8-15]. Our previous study investigating the localisation of these growth factor receptors, demonstrated expression of TGF RII, FGF R3 and IGF RI in the endothelial cells of blood vessels, as well as the native IVD cells. This suggests that the addition of such growth factors may induce blood vessel ingrowth, which could be detrimental in any treatment, because it has been reported that this is also accompanied by nerve ingrowth [16]. In contrast BMP RII expression was not observed in blood vessels suggesting that growth factors which utilise these receptors (i.e. BMPs and CDMPs) may be preferable agents for the regeneration of disc matrix in disc degeneration [17].

Two growth factors thought to stimulate proteoglycan synthesis in chondrocyte-like cells are CDMP 1 and CDMP 2 also known as BMP 14 and BMP 13 or growth and differentiation factor (GDF) 5 and 6, respectively. The distribution and effects of these growth factors have been studied in human articular cartilage *in vitro *[18,19]. In addition, the effect of these growth factors in animal models of IVD degeneration has also been studied but their expression in or effect on human IVD cells is still not fully understood [9,20-22].

Here we investigated the expression and localisation of CDMP 1 and 2 in non-degenerate and degenerate human IVDs to ascertain how their expression alters with IVD degeneration. We have previously investigated the expression of the CDMP receptor and here we relate the expression and distribution of CDMP to that seen previously for the receptor BMP RII [17]. Furthermore, the effect of CDMP 1 on cell proliferation, aggrecan and collagen type II gene expression and proteoglycan production in human NP cells derived from degenerate discs was also investigated.

## Materials and methods

### Tissue samples

Human IVD tissue was obtained either during surgery or post mortem examination with informed consent of the patient or relatives. Local research ethics committee approval was given for this work by the following Local Research Ethics Committees: Salford and Trafford, Bury and Rochdale, Central Manchester and Her Majesty's coroner.

#### Post mortem tissue

Discs recovered from patients within 18 hours of death consisted of full thickness wedges of IVD of 120° arc removed anteriorly. This allowed well-orientated blocks of tissue incorporating AF and NP to be cut for histological study. Patients with a history of sciatica sufficient to warrant seeking medical opinion, were excluded from the study.

#### Surgical tissue

Patients were selected on the basis of IVD degeneration diagnosed by magnetic resonance imaging and progression to anterior resection either for spinal fusion or disc replacement surgery for chronic LBP. Patients experiencing classical sciatica were excluded from the study. Some patients underwent fusion at more than one disc level because of spinal instability. Occasionally the specimens retrieved from multilevel fusion included discs with low (0 to 3 [see below for details of the scoring system]) histological scores (i.e. morphologically normal) at one level (Table 1). Wedges of disc tissue were removed in a manner similar to that described for cadavers.

**Table 1:** Patient details and grades of tissues used for immunohistochemistry analysis

Source	Age (years)	Clinical diagnosis	Disc level	Histological grade
Surgical	15	Normal	L4/5	0
Surgical	41	Normal	L5/S1	0
Surgical	44	Normal	L4/5	0
Surgical	41	Normal	L4/5	0
Surgical	41	Normal	L5/S1	0
Surgical	33	Disc degeneration	L4/5	1
Surgical	20	Disc degeneration	L5/S1	2
Surgical	44	Disc degeneration	L4/5	2
Surgical	47	Disc degeneration	L4/5	2
Surgical	40	Disc degeneration	L4/5	2
Surgical	39	Disc degeneration	L5/S1	5
Surgical	25	Disc degeneration	L4/5	5
Surgical	40	Disc degeneration	L4/5	6
Surgical	25	Disc degeneration	L5/S1	6
Post mortem	47	No data	L4/5	6
Surgical	43	Disc degeneration	L4/5	7
Surgical	37	Disc degeneration	L4/5	7
Surgical	55	Disc degeneration	L5/S1	7
Post mortem	Not Known	No Data	L4/5	7
Surgical	44	Disc degeneration	L5/S1	8
Surgical	33	Disc degeneration	L4/5	9
Surgical	46	Disc degeneration	L4/5	9
Surgical	37	Disc degeneration	L4/5	9
Surgical	56	Disc degeneration	L4/5	9
Surgical	33	Disc degeneration	Unknown	9
Surgical	68	Disc degeneration	L5/S1	10
Surgical	32	Disc degeneration	L4/5	10
Surgical	45	Disc degeneration	L5/S1	10
Surgical	52	Disc degeneration	Unknown	10
Surgical	45	Disc degeneration	L4/5	11

#### General procedure for tissue specimens

A block of tissue, incorporating AF and NP in continuity was fixed in 10% neutral buffered formalin, decalcified in EDTA and processed into paraffin wax. Sections were taken for H&E staining to score the degree of morphological degeneration according to previously published criteria [23]. A score of 0 to 3 represents a histologically normal (non-degenerate) disc, 4 to 8 indicates evidence of intermediate degeneration and 9 to 12 indicated severe degeneration. From this histological scoring, 30 discs were selected to represent a range of scores from non-degenerate (grades 1 to 3) up to the most severe level of histological degeneration (grade 12).

### Localisation of CDMP 1 and 2

Immunohistochemistry (IHC) was used to localise the growth factors CDMP 1 and 2 within the 30 disc samples (Table 1). The IHC protocol followed was as previously published [6]. Briefly, 4 μm paraffin sections were dewaxed, rehydrated and endogenous peroxidase blocked using hydrogen peroxide. After washing in distilled water sections were treated with chymotrypsin enzyme antigen retrieval system (0.01% w/v chymotrypsin (Sigma, Gillingham, Dorset, UK) for 20 minutes at 37°C). Following washing, non-specific binding sites were blocked at room temperature for 45 minutes in 25% w/v donkey serum in 1% w/v BSA (Sigma, Gillingham, Dorset, UK). Sections were incubated overnight at 4°C with goat polyclonal primary antibodies against human CDMP 1 (1:200 dilution, SantaCruz biotechnology, SantaCruz, California, USA) and CDMP 2 (1:500 dilution, SantaCruz biotechnology, SantaCruz, California, USA). Negative controls in which goat immunoglobulin (Ig) Gs (Dako, Ely, Cambridgeshire, UK) replaced the primary antibody (at an equal protein concentration) were used.

After washing, sections were incubated in a 1:300 dilution of biotinylated donkey anti-goat antiserum (SantaCruz biotechnology, SantaCruz, California, USA) for 30 minutes at room temperature. Disclosure of secondary antibody binding was by the streptavidin-biotin complex (Dako, Ely, Cambridgeshire, UK) technique with 3,3'-diaminobenzidine tetrahydrochloride solution (Sigma, Gillingham, Dorset, UK). Sections were counterstained with Mayers Haematoxylin (Raymond A Lamb, Eastbourne, East Sussex, UK), dehydrated and mounted in XAM (BDH, Poole, UK).

#### Image analysis

All slides were visualised using Leica RMDB research microscope and images captured using a digital camera and Bioquant Nova image analysis system (BIOQUANT Image Analysis Corporation, Nashville TN, USA). Each section was divided into three areas for analysis: the NP, inner annulus fibrosus (IAF) and outer annulus fibrosus (OAF) and analysed separately. Within each area 200 cells were counted and the number of immunopositive cells expressed as a proportion of this. Averages and standard deviations were calculated for disc sections grouped with the scores 0 to 3, 4 to 8 and 9 to 12. Data was then presented as means ± standard errors.

#### Statistical analysis

Data was non-parametric and thus Kruskal Wallis with all pair-wise comparisons *post hoc *test Conover-Inman was used to compare the numbers of immunopositive cells in degenerate groups (4 to 8, and 9 to 12) to non-degenerate discs (scores 0 to 3). These tests were performed for each area of the disc analysed (i.e. NP, IAF and OAF). In addition Wilcoxon paired samples tests were used to compare proportions of immunopositive cells in the different areas of the discs (i.e. NP v/s IAF, NP v/s OAF and IAF v/s OAF). This analysis was performed using all disc sections regardless of level of degeneration.

### Effect of CDMP on human NP samples in alginate culture

#### Isolation of disc cells

Samples of degenerate IVD tissue were obtained from three patients undergoing surgery for disc replacement for the treatment of chronic LBP (75-year-old male (Grade 7); 37-year-old female (Grade 9); and 35-year-old female (Grade 10)). NP tissue was separated and finely minced and digested with 2 U/ml protease (Sigma, Gillingham, Dorset, UK) in DMEM + F12 media for 30 minutes at 37°C and washed twice in DMEM + F12. NP cells were isolated in 0.4 mg/ml collagenase type 1 (Gibco, Paisley, UK) for four hours at 37°C.

#### Alginate culture

It is well recognised that cells derived from IVDs change their morphology and phenotype in monolayer culture becoming similar to fibroblasts. However, culturing the cells in systems such as alginate can restore the IVD cell phenotype [24]. We therefore used cells in alginate beads to investigate the effects of CDMP on cell proliferation, gene expression for aggrecan and type II collagen and proteoglycan production. Following isolation, cells were expanded in monolayer culture for two weeks prior to trypsinisation and resuspension in 1.2% w/v medium-viscosity sodium alginate (Sigma, Gillingham, Dorset, UK) in 0.15 M NaCl at a density of 4 × 10^6 ^cells/ml and alginate beads polymerised via extrusion through a 19-gauge needle into 200 mM CaCl_2_. Following washes in 0.15 M NaCl beads were transferred to culture plates and 2 ml of complete culture medium was then added to each well and cultures maintained at 37°C in a humidified atmosphere containing 5% CO_2_.

#### Treatment of cells with CDMP

Following one week in this culture system, cells were treated for two weeks with either 0 ng/ml or 10 ng/ml CDMP 1 (Autogen Bioclear, Wiltshire, UK); all treatments were performed six times. Media was changed and CDMP replaced every 48 hours. Conditioned media at each media change was frozen at -20°C for further analysis.

#### Papain digest and DMMB assay

Following treatments, triplicate samples (six beads per sample) were used for quantification of DNA and glycosaminoglycans (GAG) content using the pico green assay (Invitrogen, Paisley, UK) and the dimethylmethylene blue (DMMB) assay.

The beads were solubilised by incubation for 20 minutes at 4°C in dissolving buffer containing 55 mM sodium citrate, 30 mM Na_2_EDTA and 0.15 M NaCl, pH 6.8. The resulting suspension was subjected to mild centrifugation (100 g for 10 minutes) to separate the cells and their associated matrix in the pellet (cell-associated matrix (CM) compartment) from molecules derived from the matrix further removed from the cell surface in the supernatant (further removed matrix (FRM) compartment) as described previously [25]. The fractions were separated into fresh tubes and digested overnight at 60°C in 500 μl 20 mM sodium phosphate buffer (pH 6.8) containing 1 mM EDTA, 2 mM dithiothereitol and 100 units of papain (Sigma, Gillingham, Dorset, UK). DMMB assay was then performed using 25 μl of shark chondrotin sulphate (Sigma, Gillingham, Dorset, UK) standards (62.5 μg/ml, 31.25 μg/ml, 15.625 μg/ml, 7.81 μg/ml, 3.9 μg/ml and 0 μg/ml), 5 μl papain digested CM samples or 5 μl papain digested FRM samples or 50 μl conditioned media collected at each media change. Each sample was applied in duplicate in separate wells of a 96-well plate and 200 μl of DMMB colour regent (as described previously [26]) was added to each well. Following mixing, absorbance at *A*_525 nm _was read immediately using a Titertex Multiscan^® ^MC (Thermo Fisher, Paisley, UK). The concentration of GAGs present within each sample and total GAGs accumulated in the media over the two weeks was calculated. DNA from papain digests of cell-associated fractions were assayed along with calf thymus DNA standards using the Pico Green DNA quantification kit as per manufactures' instructions. GAG concentration was then normalised to DNA content per bead and means and standard errors calculated. In addition DNA content per bead was calculated as an indication of cell proliferation.

#### RNA extraction, and reverse transcription

Following treatments, triplicate alginate bead samples (six beads per sample) were used for analysis of aggrecan and type II collagen gene expression. RNA was extracted using TRIzol^® ^l reagent (Gibco, Paisley, UK). Prior to TRIzol^® ^extraction, alginate constructs were washed in 0.15 M NaCl and dissolved in dissolving buffer (55 mM sodium citrate, 30 mM EDTA, 0.15 M NaCl; pH 6) at 37°C for 15 minutes and then digested in 0.06% w/v collagenase type I (Gibco, Paisley, UK) for 30 minutes to allow digestion of matrix. Following RNA extraction, reverse transcription was performed using avian myeloblastosis virus reverse transcriptase (Roche, East Sussex, UK).

#### Real-time PCR

Real-time PCR was used to investigate the effects of CDMP on aggrecan (FP: 3'CCG TGT GTC CAA GGA GAA GG 5'; probe: 3'FAM- CTG ATA GGC ACT GTT GAC - MGB 5'; RP: 3' GGG TAG TTG GGC AGT GAG AC 5') (Accession numbers: [GenBank:NM_001135.2] (variant 1) and [GenBank:NM_013227.2] (variant 2) primers recognise both variants; Applied Biosystems, Warrington, UK) and type II alpha 1 collagen (FP: 3' ATG GAG ACT GGC GAG ACT TG 5'; probe: 3' FAM - CCC AAT CCA GCA AAC G - MGB 5'; RP: GCT GCT CCA CCA GTT CTT 5') (Accession numbers: [GenBank:NM_001844.4] (variant 1) and [GenBank:NM_033150.2] (variant 2) primers recognise both variants; Applied Biosystems, Warrington, UK) gene expression using 18 s as the housekeeping gene (PDAR: Applied Biosystems, Warrington, UK) and genomic DNA standard curves to generate copy number per 100 ng cDNA as described previously [27].

### Statistical analysis

Mann Whitney U tests were used to compare untreated and CDMP-treated samples to investigate significant differences in DNA content, GAG content and release into media and aggrecan and type II collagen gene expression.

## Results

### Immunohistochemical localisation of CDMP 1 and CDMP 2

**Figure 1:**
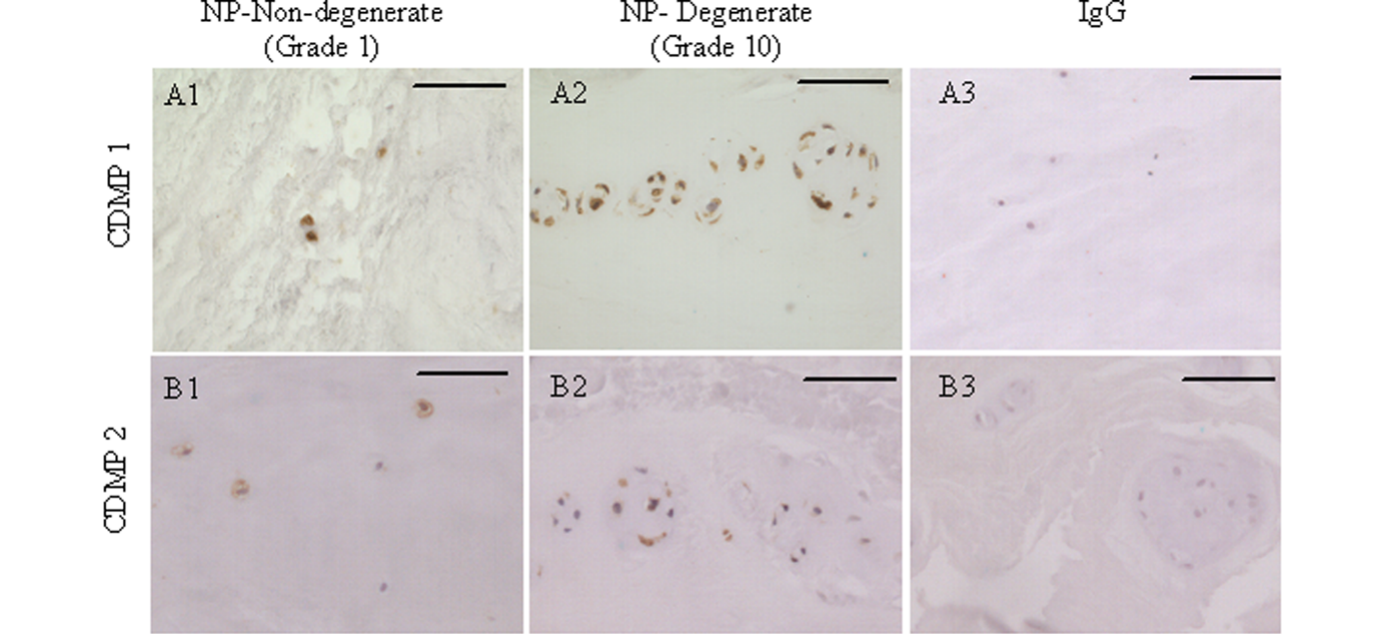
Examples of immunohistochemical staining for CDMPs in human intervertebral disc. (row A) Cartilage derived morphogenetic protein (CDMP 1) and (row B) CDMP 2. Images are of nucleus pulposus of grade 1 non-degenerate discs (column 1), the nucleus pulposus of grade 10 degenerate discs (column 2) and IgG controls for each antibody. Bars = 570 μm.

**Figure 2:**
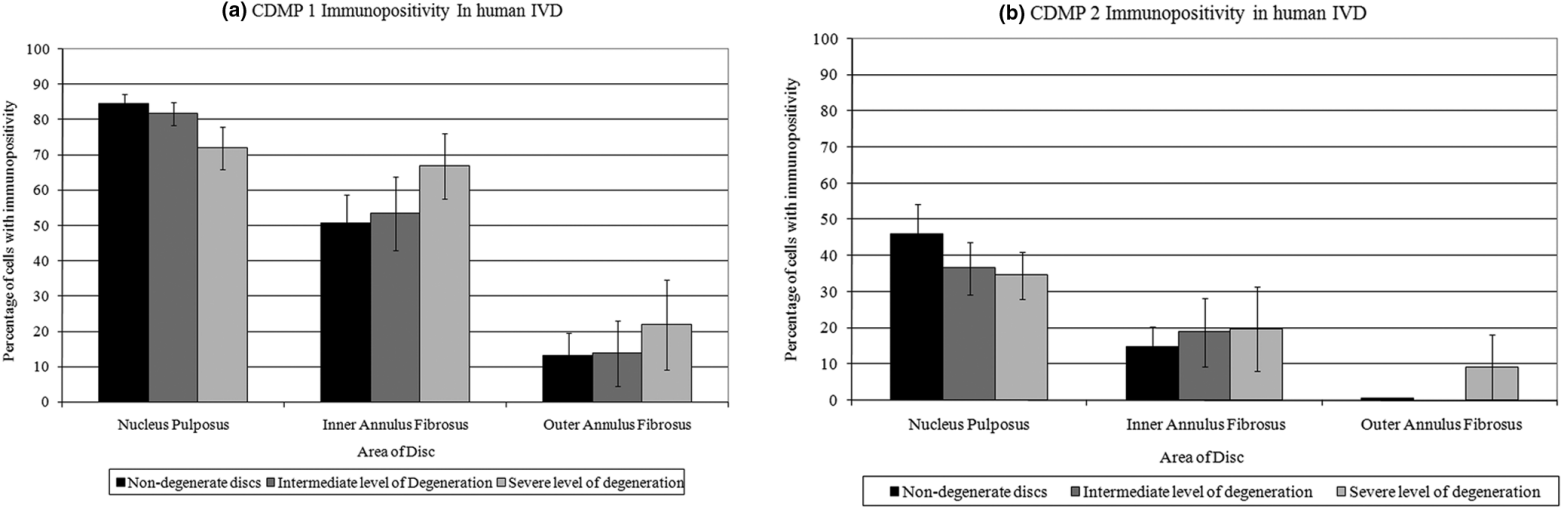
Assessment of immunopositive staining for CDMP 1 and 2 in human intervertebral discs. Percentage of cells with immunopositivity for **(a) **cartilage derived morphogenetic protein (CDMP) 1, **(b) **CDMP 2, according to location in the disc and grade of intervertebral disc degeneration (n = 30). Data are presented as means ± standard error.* *P *< 0.05 compared with non-degenerate discs.

Immunopositive staining for both CDMP 1 and CDMP 2 was restricted to the cytoplasm of native disc cells in both non-degenerate and degenerate discs and there was no statistical significance between non-degenerate and degenerate discs (*P *> 0.05; Table 2). Staining was particularly prominent in the cytoplasm of the chondrocyte-like cells of the NP and IAF, with both single cells and those in clusters showing immunopositivity (Figures 1 and 2). CDMP 1 immunopositivity was observed in a higher proportion of cells in both non-degenerate and degenerate discs than CDMP 2 (*P *< 0.05). A greater proportion of cells were immunopositive for CDMP 1 and CDMP 2 in the NP than the IAF (*P *< 0.05), and the proportion of immunopositive cells in the OAF was always lower than that seen in the NP and IAF (all targets *P *< 0.05). No immunopositivity was observed in the matrix of the IVD or in the endothelial cells of the blood vessels for either CDMP 1 or 2. IgG controls were negative (Figure 1).

**Table 2:** Analysis of immunohistochemical data: *P *values for analysis of CDMP1 and 2 expression in different areas of disc in non-degenerate v/s degenerate discs

IVD area analysed for CDMP expression	Intermediate degeneration (*P*)	Severe degeneration (*P*)
CDMP 1 expression in NP Non-degenerate v/s degenerate	0.302	0.106
CDMP 1 expression in IAF Non-degenerate v/s degenerate	0.336	0.112
CDMP 1 expression in OAF Non-degenerate v/s degenerate	0.461	0.362
CDMP 2 expression in NP Non-degenerate v/s degenerate	0.241	0.124
CDMP 2 expression in IAF Non-degenerate v/s degenerate	0.479	0.521
CDMP 2 expression in OAF Non-degenerate v/s degenerate	0.679	0.465

CDMP = cartilage derived morphogenetic protein; IAF = inner annulus fibrosus; IVD = intervertebral disc; NP = nucleus pulposus; OAF = outer annulus fibrosus.

### Immunohistochemical staining for BMP RII

We have previously shown BMP RII immunopositive staining in the human IVD with a greater number of immunopositive cells within the NP than the IAF and OAF (*P *< 0.05). Furthermore, in IVDs graded as intermediate degeneration there was an increase in the proportion of immunopositive cells, which was significant in the NP (*P *< 0.05) [17]

### Effect of CDMP 1 on proliferation of human NP cells derived from degenerate discs

**Figure 3:**
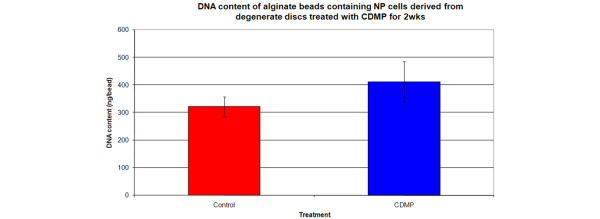
Effect of CDMP treatment on DNA content of alginate beads containing NP cells derived from degenerate discs treated with CDMP for two weeks. Data are presented as means ± standard error. CDMP = cartilage derived morphogenetic protein; NP = nucleus pulposus.

To determine the effect of CDMP 1 on cellular proliferation DNA content per alginate bead was calculated following two weeks of treatment with CDMP. An increase in DNA content (28% increase in CDMP-treated cells v/s untreated cells) was observed in the alginate bead cultures treated with CDMP but this did not reach significance (*P *= 0.35; Figure 3).

### Effect of CDMP 1 on GAG production of human NP cells derived from degenerate discs

**Figure 4:**
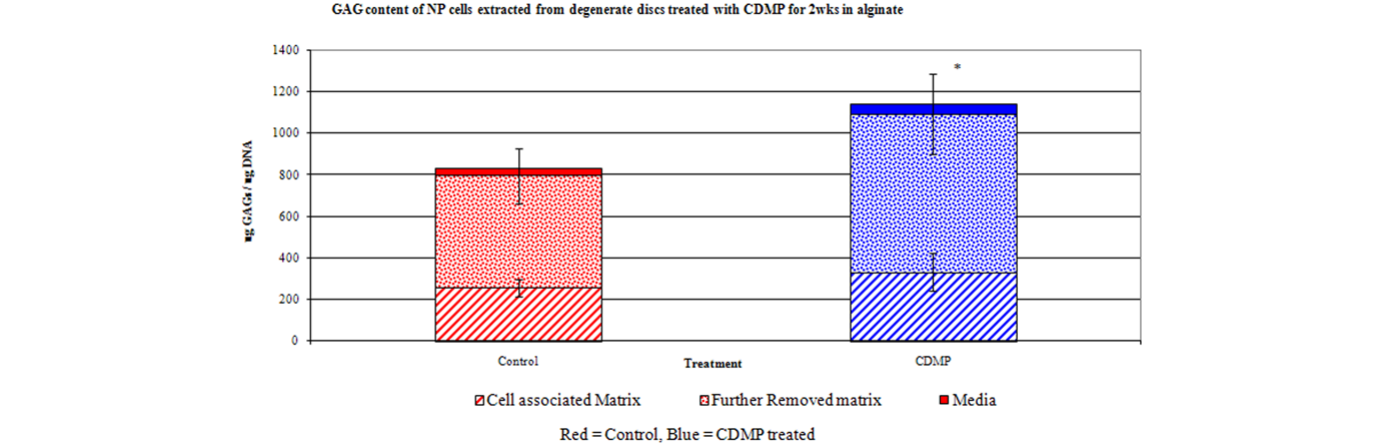
Effect of CDMP treatment on GAG content of NP cells derived from degenerate discs. Data are presented as GAG content of the cell associated matrix, further removed matrix and GAG released into the media per ug DNA (means ± standard error. * *P *< 0.05 compared with untreated controls). CDMP = cartilage derived morphogenetic protein; GAG = glycosaminoglycan; NP = nucleus pulposus.

A significant increase in overall GAG production (i.e. within the CM, FRM and media together) was observed in NP cells derived from degenerate discs treated with 10 ng/ml CDMP 1 for two weeks compared with untreated NP cells (*P *< 0.05). An increase in GAG content of CM in CDMP-treated cultures was observed but this did not reach significance (*P *= 0.43). However, the GAG content within the FRM was significantly increased following CDMP 1 treatment for two weeks (*P *< 0.05). No difference was observed in the GAG released into the media during the two weeks treatment with CDMP from untreated alginate bead cultures of NP cells derived from degenerate discs (*P *= 0.24; Figure 4).

### Effect of CDMP 1 on gene expression for aggrecan and collagen type II in human NP cells derived from degenerate discs

**Figure 5:**
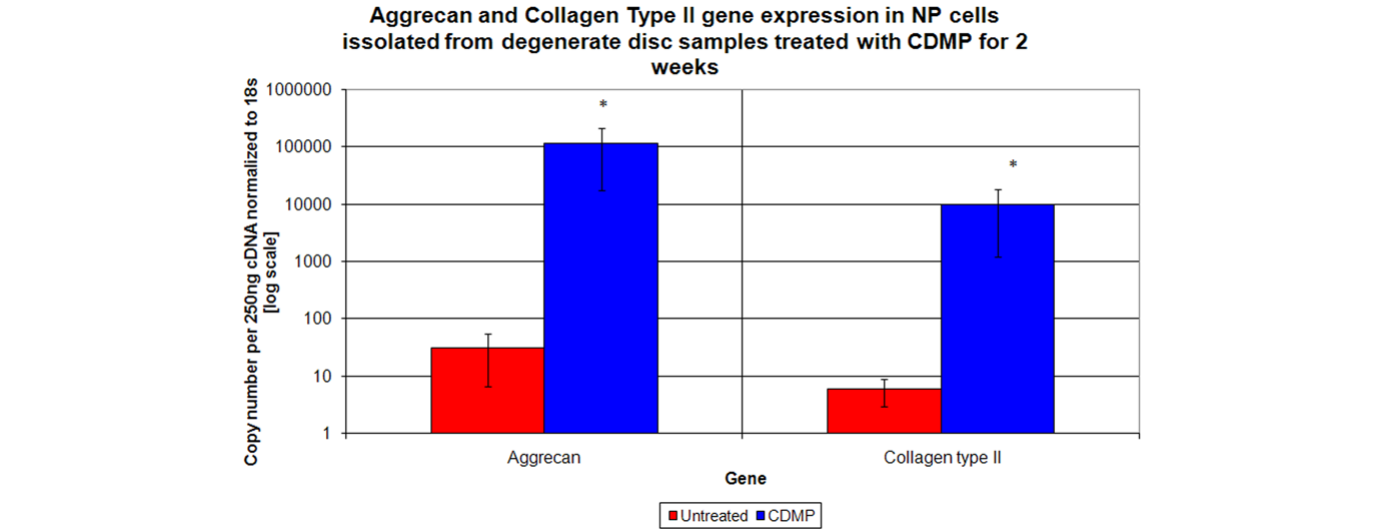
Effect of CDMP treatment on aggrecan and type II collagen gene expression in NP cells derived from degenerate discs treated with CDMP for two weeks. Absolute quantification of copy number per 250 ng cDNA normalized to the housekeeping gene 18 s. Data are presented as means ± standard error. * *P *< 0.05 compared with untreated controls. CDMP = cartilage derived morphogenetic protein; NP = nucleus pulposus.

A significant increase in both aggrecan (3831-fold increase) and collagen type II (1660-fold increase) gene expression was observed in NP cells derived from degenerate discs cultured in alginate beads and treated with 10 ng/ml CDMP 1 for two weeks (*P *< 0.05; Figure 5).

## Discussion

A major cause of LBP is degeneration of the IVD, of which proteoglycan loss is a key feature and has been linked to loss in disc height, de-stabilisation of the motion segment and the ingrowth of blood vessels and nerves resulting in generation of pain [28,29]. Thus a potential therapeutic approach to repair the degenerate disc would be the stimulation of normal disc matrix production particularly increased synthesis of proteoglycans. A number of growth factors have been suggested as possible therapeutic agents. However, our previous study suggested that the addition of growth factors which bound to TGF RII, FGF R3 and IGF RI may also induce unwanted blood vessel ingrowth [17]. However, we demonstrated that growth factors, such as CDMP 1 and 2, which elicit their response via BMP RII, should not induce blood vessel ingrowth.

Here we demonstrate the synthesis and localisation of CDMP 1 and CDMP 2 within human IVDs. Although a small decrease in the proportion of cells within the NP staining for CDMP 1 and CDMP 2 was observed during degeneration this was not significant. Similarly Bobacz and colleagues demonstrated that both CDMP 1 and CDMP 2 were expressed in normal and osteoarthritic (OA) articular cartilage with no change seen during OA [18]. This suggests that the pathogenesis of disc degeneration or OA is not associated with a reduced expression of these growth factors.

CDMP has been shown to result in increased proteoglycan production in human mesenchymal stem cells [30], a chondrocyte cell line [31], and in human articular chondrocytes [18,19]. Recently, a small number of studies have also demonstrated proteoglycan stimulation in bovine, rabbit and mouse disc cells [21,22]. However, to date, no studies have demonstrated an increase in proteoglycan production in degenerate human IVD cells following CDMP treatment. Here we investigated the effect of CDMP 1 on human NP cells cultured in an alginate bead system. Importantly an alginate bead culture system was used as this maintains the *in vivo *phenotype of IVD cells, which is lost in monolayer culture [25,32]. Our results demonstrate that cells derived from degenerate human discs can also respond to CDMP with an increase in GAG production, although our study only used three patient samples. These results confirm those derived from animal disc cells where CDMP resulted in significant increases in GAG production [21,22]. The accumulation of GAG within alginate beads was investigated within the compartments: CM and FRM, together with GAG released into media. The majority of the GAG produced by the degenerate NP cells was found in the FRM, and this was the area which showed a significant increase in GAG accumulation following treatment with CDMP 1. The CM is thought to represent the highly structured compartment encircling each cell and corresponds to the combined pericellular and territorial matrix pools which surround each cell *in vivo *[25,33,34]. In contrast the more loosely organised compartment known as the FRM, accounting for approximately 95% of the total volume of matrix, is thought to represent the interterritorial matrix compartment *in vivo *[25,34]. As this area is thought to account for the majority of the matrix *in vivo *the fact that more GAGs were found in this area of matrix following stimulation with CDMP is promising for future therapeutic approaches.

The current study also showed that CDMP1 induced dramatic increases in the gene expression for the matrix molecules aggrecan and collagen type II within degenerate human NP cells, as has been reported in mouse IVD cells [22]. During disc degeneration the production of both aggrecan and collagen type II is decreased [23,35] leading to reduced hydration and ability to withstand load. Thus, if a growth factor could be applied which can successfully stimulate the synthesis of these important matrix molecules this would be of benefit for regenerating the degenerate disc.

Previous studies investigating the effect of CDMP1 on rabbit disc cells in monolayer [9] and mouse [22] and bovine disc cells in alginate [21] have shown significant increases in cell proliferation. Here we showed a small increase in proliferation of human disc cells in alginate culture following treatment with CDMP1 for two weeks, although, possibly due to the small sample size, this did not reach significance. Increases in proliferation could be of benefit in a therapeutic approach as a mechanism to replace some of the cells lost through apoptosis and senescence which are common features during disc degeneration [27,36].

Importantly, this study, together with previous animal studies, suggests CDMP could be a useful therapeutic agent in the regeneration of the degenerate IVD and provides supporting evidence for the clinical use of CDMP in human IVD degeneration. Indeed a phase I/II clinical trail has just started investigating the efficacy and safety of recombinant GDF 5 (CDMP 1) injection into the IVD for degenerative disc disease [37]. However, it must be noted that any proposed therapy may have to target a number of other problems that are associated with disc degeneration. Combinations of factors may be needed in order to promote matrix synthesis and inhibit the increased catabolism seen within the degenerate disc [38,39]. Furthermore, it has been shown that the nutrient supply diminishes with degeneration, which may also limit disc cell self-renewal and function [40]. Thus, potential therapeutic growth factors may have to be combined with therapies aimed at restoring disc nutrition or targeted at those patients in which the cartilaginous endplates (through which nutrients are received) are unaffected, that is not calcified, or sclerotic [40].

## Conclusions

Our data demonstrates that CDMP 1 and 2 protein is expressed by both non-degenerate and degenerate discs together with its receptor (BMP RII), suggesting CDMP is involved in the normal matrix homeostasis with the human IVD. Importantly we have demonstrated, for the first time, that human disc cells derived from degenerate discs retain their ability to respond to CDMP and that such treatment leads to an increase in aggrecan and collagen type II gene expression and increased accumulation of GAGs. Together this data suggests that CDMP is an important anabolic growth factor in the IVD and could be a suitable therapy to aid in IVD repair/regeneration, via stimulation of matrix synthesis.

## Abbreviations

AF: annulus fibrosus; BMP: bone morphogenetic protein; BMP RII: BMP receptor 2; BSA: bovine serum albumin; CDMP: cartilage derived morphogenetic protein; CM: cell-associated matrix; DMEM: Dulbecco's modified eagle medium; DMMB: dimethylmethylene blue; FGF: fibroblast growth factor; FGF R3: FGF receptor 3; FRM: further removed matrix; GAGs: glycosaminoglycans; GDF: growth differentiation factor; H&E: haematoxylin and eosin; IAF: inner annulus fibrosus; Ig: immunoglobulin; IGF: insulin-like growth factor; IGF RI: IGF receptor 1; IHC: immunohistochemistry; IVD: intervertebral disc; LBP: low back pain; MMP: matrix metalloproteinase; NP: nucleus pulposus; OA: osteoarthritis; OAF: outer annulus fibrosus; PCR: polymerase chain reaction; TGF: transforming growth factor; TGF RII: TGF receptor 2.

## Competing interests

The authors declare that they have no competing interests.

## Authors' contributions

CLM helped conceive the study, participated in its design, performed the majority of the laboratory work and all the analysis and co-wrote the manuscript. AJF participated in interpretation of data and contributed to the preparation of the final manuscript. JAH conceived the study, secured funding, contributed to its design and co-ordination, participated in interpretation of data and contributed to the preparation of the final manuscript. All authors read and approved the final manuscript.
